# Styrene-Based Elastomer Composites with Functionalized Graphene Oxide and Silica Nanofiber Fillers: Mechanical and Thermal Conductivity Properties

**DOI:** 10.3390/nano10091682

**Published:** 2020-08-27

**Authors:** Jaehyeung Park, Jaswinder Sharma, Kyle W. Monaghan, Harry M. Meyer, David A. Cullen, Andres M. Rossy, Jong K. Keum, David L. Wood, Georgios Polizos

**Affiliations:** 1 Energy and Transportation Science Division, Oak Ridge National Laboratory, Oak Ridge, TN 37831, USA; parkj@knu.ac.kr (J.P.); sharmajk@ornl.gov (J.S.); wooddl@ornl.gov (D.L.W.III); 2 Department of Bio-Fibers and Materials Science, Kyungpook National University, Daegu 41566, Korea; 3 Materials Science and Technology Division, Oak Ridge National Laboratory, Oak Ridge, TN 37831, USA; kylewmonaghan@gmail.com (K.W.M.); marquezae@ornl.gov (A.M.R.); 4 Center for Nanophase Materials Sciences, Oak Ridge National Laboratory, Oak Ridge, TN 37831, USA; meyerhmiii@ornl.gov (H.M.M.III); cullenda@ornl.gov (D.A.C.); 5 Neutron Scattering Division, Oak Ridge National Laboratory, Oak Ridge, TN 37831, USA; keumjk@ornl.gov

**Keywords:** SBR composites, SBS composites, graphene oxide, silica nanofibers, mechanical properties, thermal conductivity

## Abstract

The mechanical and thermal conductivity properties of two composite elastomers were studied. Styrene–butadiene rubber (SBR) filled with functionalized graphene oxide (GO) and silica nanofibers, and styrene–butadiene–styrene (SBS) block copolymers filled with graphene oxide. For the SBR composites, GO fillers with two different surface functionalities were synthesized (cysteamine and dodecylamine) and dispersed in the SBR using mechanical and liquid mixing techniques. The hydrophilic cysteamine-based GO fillers were dispersed in the SBR by mechanical mixing, whereas the hydrophobic dodecylamine-based GO fillers were dispersed in the SBR by liquid mixing. Silica nanofibers (SnFs) were fabricated by electrospinning a sol–gel precursor solution. The surface chemistry of the functionalized fillers was studied in detail. The properties of the composites and the synergistic improvements between the GO and SnFs are presented. For the SBS composites, GO fillers were dispersed in the SBS elastomer at several weight percent loadings using liquid mixing. Characterization of the filler material and the composite elastomers was performed using x-ray photoelectron spectroscopy, x-ray diffraction, transmission electron microscopy, scanning electron microscopy, thermogravimetric analysis, dynamic mechanical analysis, tensile testing, nanoindentation, thermal conductivity and abrasion testing.

## 1. Introduction

Composite elastomers are a very significant category of polymeric materials with numerous applications such as tire manufacturing, high-performance elastomers, gas barrier materials and advanced binders for energy storage devices [1,2,3,4,5,6,7]. The incorporation of filler material in the elastomer matrix can provide significant reinforcement [8,9] and impart new properties to the elastomer such as thermal and electrical conductivity [10,11,12,13]. Tailoring the filler-elastomer interactions and optimizing the dispersion of the filler material is imperative for the fabrication of high-performance elastomers. Graphene oxide (GO) is one of the most promising filler materials that can potentially replace the carbon black and silica nanoparticles that are commonly been used in the fabrication of rubber composites [14,15]. Their planar structure, high surface area when exfoliated, and the possibility to tailor the surface functionality makes them unique fillers. Moreover, the strong interaction between styrene groups and two-dimensional carbon nanomaterials [16,17] makes GO an excellent filler for styrene-based elastomers [18,19].

Herein, we report our studies on non-crosslinked styrene–butadiene rubber (SBR) and styrene–butadiene–styrene (SBS) composites. The focus of this work is to investigate the effect of the different functionalities and mixing conditions on the mechanical and thermal conductivity properties of the composites. Tailoring the surface functionality and wettability of the GO fillers is very important as it can result in better interfacial interactions between the GO and the elastomer and therefore in better dispersion of the GO. Sulfur groups are commonly being used to functionalize GO in order to form covalent bonds with the butadiene groups of the SBR. The intrinsic hydrophilicity of the GO due to the high oxygen content and the hydrophilic nature of the sulfur groups make the exfoliation and dispersion of the GO in the hydrophobic SBR matrix more difficult. Strongly reduced GO has significantly lower oxygen content that is typically less than 10 at.%. Such low oxygen content can limit the amount of functionality that can be grafted on the GO surface and can also hinder the exfoliation of the GO layers due to the stronger van der Waals forces between GO layers. The oxygen content of the GO or reduced GO must be optimized depending on the application [20]. In this study, we used GO with low oxygen content. Functionalization with cysteamine groups and the simultaneous reduction [21,22,23] of the GO was performed to incorporate sulfur groups and partially reduce the oxygen content of the GO fillers. The hydrophilic cysteamine-modified GO fillers were dispersed in the hydrophobic SBR using mechanical mixing. A different surface functionality was also studied. The hydrophilic GO fillers were functionalized with dodecylamine to covalently bond hydrophobic alkyl groups on the GO surface and improve their dispersion in nonpolar solvents. A liquid mixing technique was used to disperse the dodecylamine-modified GO fillers in SBR.

A hybrid composite was also fabricated based on the GO with the surface functionality that resulted in better reinforcement and a high aspect ratio hydrophobic silica nanofibers (SnFs). The high surface area of the GO fillers limits the amount of GO that can be effectively dispersed in the elastomer. The addition of a filler with complementary properties, such as SnFs, can synergistically improve the properties of the composite. SBS–GO composites at several GO weight loadings were also studied. SBS is a block copolymer that does not require crosslinking and can be processed like a thermoplastic resin. Although it is less robust than the crosslinked SBR, the addition of GO can provide additional benefits that are associated with the significant increase in the thermal conductivity.

## 2. Experimental

Commercially available graphene oxide (GO) was functionalized with cysteamine and simultaneously reduced by adding KOH in the solution. GO was dispersed in water (20 mL solution of 1 mg mL^−1^ GO) and the pH of the solution was adjusted to 8 using KOH. The GO dispersion was mixed with cysteamine (0.25 mmol) and the mixture was then subject to ultrasonication for 30 min. The homogeneous dispersion was then vigorously stirred overnight at 80 °C. The resulting thiol-modified reduced GO (rGO–SH) powder was centrifuged and washed with distilled water and ethanol and dried.

GO fillers functionalized with dodecylamine (DA) were prepared by adding DA (0.25 mmol) in a suspension of GO in ethanol (20 mL solution with of 1 mg mL^−1^ GO). The mixture was refluxed overnight at 90 °C while stirring. To remove the physically adsorbed DA, the DA modified powder (GO–DA) was centrifuged, washed with ethanol and dried.

Silica nanofibers were fabricated by electrospinning a sol–gel precursor solution. Tetraethyl orthosilicate (TEOS, 1.6 mL) was dissolved in a solution of polyvinylpyrrolidone (0.675 g of PVP, Mw = 1,300,000 g/mol,) in ethanol (15 mL) and hydrochloric acid (0.1 mL of 2 M HCl). The solution was electrospun at flow rate of 30 μL/min and at a voltage of 16 kV. The PVP/silica nanofibers were collected on the collector that were kept at 15 cm from the needle tip. The resulting nanofibers were dried at 80 °C for 12 h. Silica nanofibers were obtained after calcination at 650 °C for 12 h. The prepared silica nanofibers were ground with a mortar and pestle and then were modified with triethoxy(octyl)silane.

Composites of SBR (Mw ~100,000 g/mol) filled with GO (4 wt.%) and SBR filled with rGO–SH (4 wt.%) were prepared by mechanical mixing. A Brabender mixer was used for the mixing of the SBR composites. The temperature of the mixer was 110 °C and the rotor speed was 90 rpm. SBR composites filled with GO–DA (4 wt.%) and octylsilane modified SnFs (15 wt.%) were prepared by solution mixing. The octylsilane modified SnFs and the GO–DA fillers were dispersed and exfoliated in toluene by ultrasonic agitation for 0.5 h. The resulting suspension was mixed with SBR that was dissolved in toluene. The SBR composite mixture was homogenized using a shear mixer at 9000 rpm at ambient temperature for 0.5 h and thereafter it was purred into vigorously stirred methanol to coagulate. The precipitate was retrieved by filtration and was dried in a vacuum oven at 80 °C for 3 days. SBR composites were compression-molded at 210 °C under 4 tons for 20 min. The mechanical properties of the GO and SnF composites were compared to an SBR composite that was filled with 9 wt.% non-functionalized silica nanoparticles (SBR/SnP). The silica nanoparticles were prepared using a sol–gel method and their size was 30–50 nm [24]. Silica nanoparticles are common reinforcement filler for the SBR and they were used for comparison against the GO and SnF fillers. The SBR/SnP composite was prepared using the same procedure that was used for the preparation of the other mechanically mixed composites. All SBR composites studied herein were not crosslinked. SBS–GO composites (SBS; Mw ~140,000 g/mol, styrene content ~30 wt.%) were prepared by solution mixing according to the procedure used for the liquid mixing of the SBR composites. The SBR composites were compression-molded in a cast (80 × 80 × 1 mm) at 150 °C with 4 tons for 20 min. Composites were prepared at 1, 3, 5 and 10 wt.% GO.

X-ray photoelectron spectroscopy (XPS) and x-ray diffraction (XRD) techniques were used to characterize the surface chemistry of the functionalized filler material. Transmission electron microscopy (TEM), scanning electron microscopy (SEM), thermogravimetric analysis (TGA), dynamic mechanical analysis (DMA), tensile, nanoindentation, thermal conductivity and Taber abrasion testing were used to study the properties of the composites. Samples were prepared for SEM and TEM measurements. For the SEM measurements, samples were sputtered with gold using a Cressington sputter coater. The sputtered samples were mounted on carbon tape. Measurements were carried out on a Zeiss Merlin microscope. For the TEM measurements, thin samples were incorporated into epoxy resin. A Leica microtome was used to slice thin sections that were mounted on lacey carbon copper TEM grids. Measurements were carried out on a Hitachi HF3300 operated at 300 kV. Details on the experimental setups are provided elsewhere [25,26,27,28]. The TGA measurements were performed in a nitrogen environment. The heating rate was 10 °C/min. The heating rate for the DMA measurements was 5 °C/min.

## 3. Results and Discussion

### 3.1. SBR Composites

**Figure 1:**
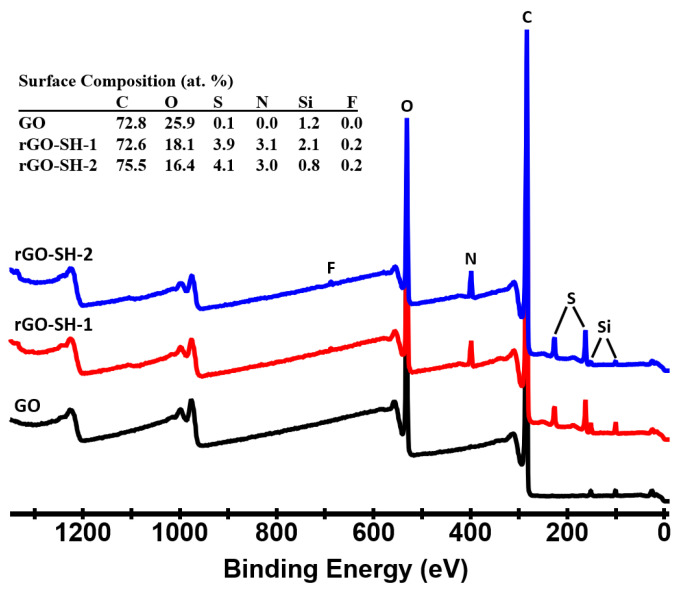
XPS survey scans and the overall surface composition of the graphene oxide (GO) and reduced graphene oxide functionalized with cysteamine (rGO–SH). Two functionalized rGO–SH samples were measured. They are designated on the plot as rGO–SH-1 and rGO–SH-2. The peaks at approximately 980 and 1200 eV are the oxygen and carbon O–KLL and C–KLL Auger peaks, respectively.

XPS was used to analyze the surface composition of the cysteamine-functionalized GO fillers. The samples were mounted onto double-sided tape and introduced into the XPS instrument through a vacuum-pumped load-lock. Survey scans were acquired to determine the elements present on the surface of the fillers. In order to access the reproducibility of the functionalization process, two functionalized rGO–SH samples were measured. These samples are encoded as rGO–SH-1 and rGO–SH-2. The survey scans and the overall surface composition according to the survey data are shown in Figure 1. The predominant elements on the surface of the samples are C, O, N, and S. The samples also showed trace amounts of F and Si. A significant amount of S and N was incorporated on the surface of the cysteamine-functionalized samples as compared to the pristine GO. The rGO–SH-1 and rGO–SH-2 samples have very similar surface composition. The functionalized samples were partially reduced. The O content was reduced to 16–18 at. % as compared to approximately 26 at. % in the pristine GO sample.

**Figure 2:**
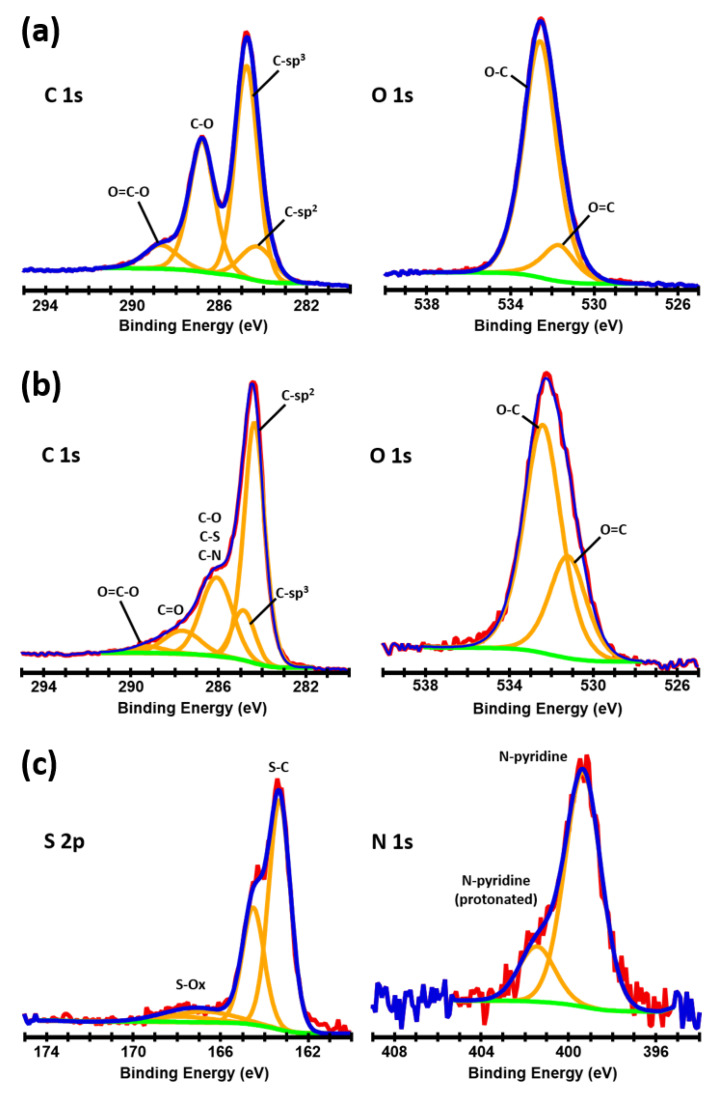
Core level spectra fitting analysis of the (**a**) GO and (**b**,**c**) rGO–SH-2 samples.

Core level spectra were acquired for the elements identified in the survey scans. The C 1s, O 1s, S 2p, and N 1s data along with the peak fit results for the GO and rGO–SH-2 samples are shown in Figure 2. The spectra of the rGO–SH-1 and rGO–SH-2 samples are similar and therefore only the rGO–SH-2 sample is presented herein. Four or five peaks were used to fit the C 1s spectra. One for each C (sp2) and C (sp3); one peak for the overlapping C–O/C–N/C–S bonds (only C–O was observed in GO); one peak for the C=O (only observed in rGO–SH-1 and rGO–SH-2); and another for the O=C–OH. Two peaks were used to fit the O 1s. One for the O–C and another for the O=C. When N was present, two peaks were used for fitting. One at ~399 eV assigned to the aniline–nitrogen and another smaller feature shifted to higher binding energy was assigned to a protonated form of aniline. S (trace amount from the oxidation process) was present in GO in the oxidized form of SOx (binding energy ~ 167 eV). In the rGO–SH-1 and rGO–SH-2 samples, the S was primarily found to be bonded to carbon (C–S–H, binding energy ~ 163 eV). The percent of each component is shown in Table 1.

**Figure 3:**
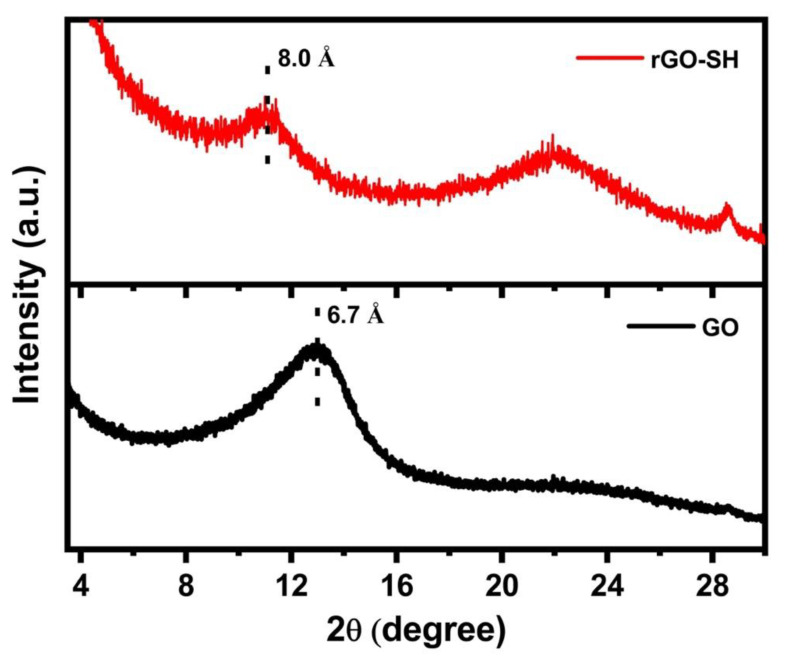
XRD patterns for the GO and rGO–SH samples. The basal plane *d*-spacing values are indicated on the plot.

According to the H–S–C values in Table 1, the cysteamine functionality on the rGO surface is approximately 3.4–3.7 at. %. The incorporated thiol (–SH) groups can promote the dispersion of the fillers by forming covalent bonds with the elastomer matrix. The increase in the interlayer spacing between the GO layers due to the intercalated cysteamine groups can also reduce the electrostatic interactions between the GO planes and assist the dispersion of the filler material in the SBR matrix. The XRD patterns of the GO and rGO–SH are shown in Figure 3. The basal plane *d*-spacing according to Bragg’s law is 6.7 and 8.0 Å for the GO and rGO–SH, respectively. The *d*-spacing slightly increased due to the intercalated functional groups [29,30] despite the O reduction in the rGO–SH, which is expected to result in stronger *π*–*π* stacking interactions and a decrease in the *d*-spacing [31]. The 2θ value of the peak that corresponds to the intercalated cysteamine is comparable to the 2θ value reported in the literature for partially reduced graphene oxide modified with cysteamine [32]. The broad peak at 22° for the rGO–SH sample in Figure 3 can be attributed to the thiolation and reduction of the GO surface [26,33]. The high intensity of the peak indicates a high thiolation degree which is in good agreement with the surface composition according to the XPS analysis. The peak at 28.6° in the rGO–SH spectra is due to the reduction of the GO. The position and shape of this peak depends on the reduction process [34,35].

**Figure 4:**
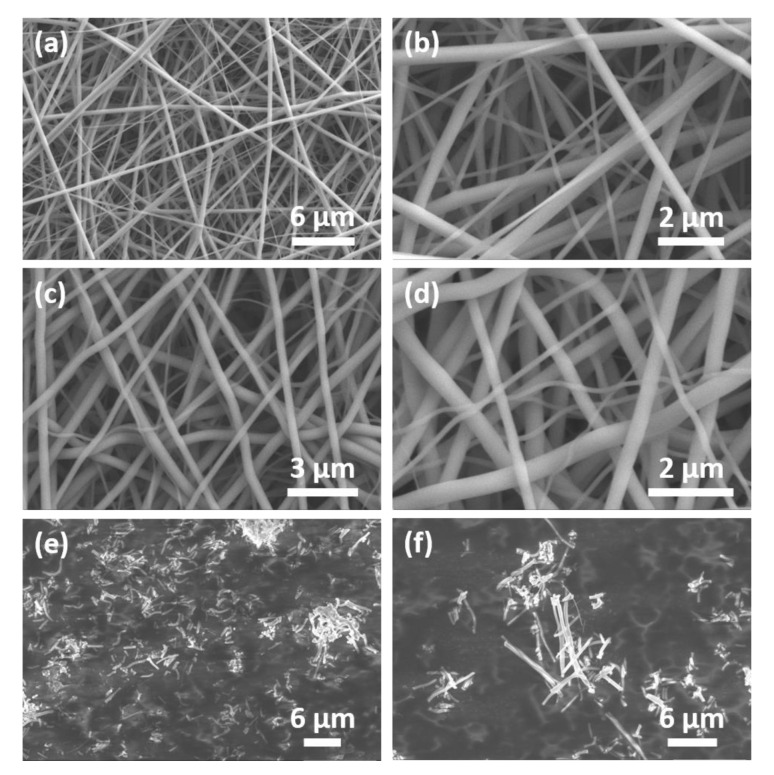
SEM images of the electrospun silica nanofibers. (**a**,**b**) polyvinylpyrrolidone (PVP)/silica nanofibers, (**c**,**d**) silica nanofibers after calcination at 650 °C for 12 h, and (**e**,**f**) ground silica nanofibers.

The electrospun silica nanofibers are shown in Figure 4. The concentration of the polymer and precursor solution as well as the electrospinning parameters were optimized to obtain continuous and uniform fibers without the formation of particles and bead-like and structures [36]. The diameter of the calcined fibers is less than 400 nm. XRD measurements (not shown here) were performed to confirm the amorphous phase of the silica fibers. The fibers were ground (Figure 4e,f) and functionalized with triethoxy(octyl)silane to better disperse in the SBR matrix. Their high aspect ratio is anticipated to result in stronger interfacial adhesion between the fibers and the elastomer and therefore to better improve the mechanical properties of the composite elastomers compared to the elastomers filled with spherical silica particles [37,38]. To predict the tensile properties of fiber reinforced composites, several models have been proposed [39,40,41,42]. According to the equation developed by Cox [31] and later modified to include randomly oriented fibers [32,34,43], the correlation between the fiber aspect ratio and the tensile modulus of the composite, *Y_c_*, is given by

(1) $$Y_{c}=\left(n_{o}n_{l}Y_{f}-Y_{m}\right)V_{f}+Y_{m}$$

In the above equation, $Y_{m}$ is the modulus of the matrix, $V_{f}$ is the volume fraction of the fibers, $n_{o}$ is the orientation factor of the fibers (with values ranging between 1 for aligned and 1/5 for the randomly oriented fibers) and $n_{l}$ is the length efficiency factor which is given by

(2) $$n_{l}=1-\frac{Tanh\left(\frac{\alpha l}{D}\right)}{\alpha l/D}$$

where:

(3) $$\alpha =\sqrt{\frac{-3Y_{m}}{2Y_{f}lnV_{f}}}$$

In Equation (2), *l* and *D* represent the length and diameter of the fiber, respectively. Based on the Equations (1)–(3), Coleman et al. [34] pointed out that fibers with a high aspect ratio are required to reinforce the modulus of the composite matrix. The length efficiency factor must be approximately 1 and the ratio *l*/*D* > 10. Similar equations can also be used to model the tensile strength of fiber composites [34]. The high aspect ratio of the synthesized SnFs is associated with the *l* and *D* values which are several micrometers (after they were ground) and less than 400 nm, respectively. However, the ground process that was performed to prevent the formation of large aggregates of entangled fibers during the mixing makes difficult the precise control of their length.

In addition to the expected improvements due to the high aspect ratio, the nanometer size diameter of the silica fibers is also very important in order to reinforce the mechanical properties of the composites. The tensile strength and modulus are not intrinsic material properties. They depend on the size and geometry of defects that are formed during the material fabrication. In order to explain the discrepancy between the theoretically predicted and the significantly lower experimental values, Griffith introduced the effective stress which is the applied stress on the defect points [44,45]. It was shown that the fracture strength, $\sigma^{f}$, can be expressed as

(4) $$\sigma^{f}=\frac{K_{c}}{{\left(\pi \lambda \right)}^{1/2}}$$

where $K_{c}$ is the fracture toughness and λ is the defect size. The above relation indicates that the strength of the fibers is inversely proportional to the square root of the defect size. Since the size of the defects decreases when the fiber diameter is decreased, it is apparent that the tensile properties of the silica nanofibers will be superior compared to the properties of fibers with a micrometer-size diameter. Silica nanowires, 100 nm in diameter, have been reported to demonstrate Young’s modulus value approximately 50 GPa [46].

**Figure 5:**
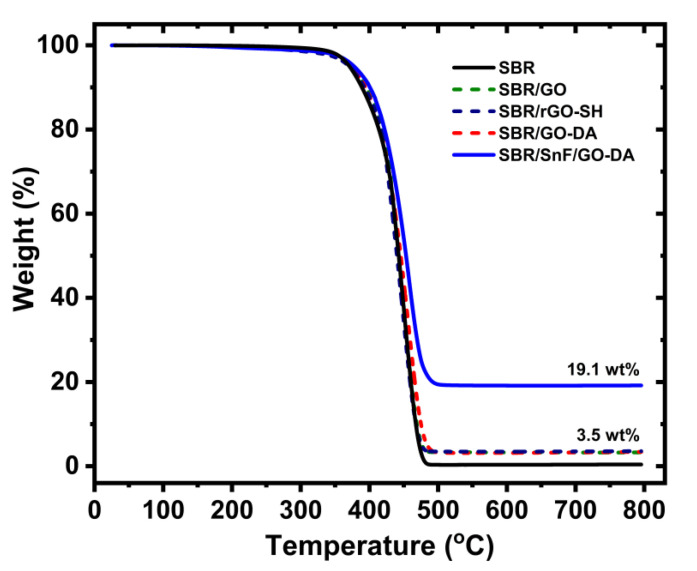
TGA measurements of the styrene–butadiene rubber (SBR) composites. The silica and GO weight percent loadings according to the plateau values are in good agreement with the filler loadings of the composites which are 15 and 4 wt.%, respectively.

SBR composites were fabricated using two mixing techniques. The SBR/GO and SBR/rGO–SH samples were prepared using mechanical mixing. The SBR/GO–DA and SBR/SnF/GO–DA samples were prepared using solution mixing followed by coagulation (rapid precipitation of the SBR composite) to prevent the agglomeration of the fillers. Modification of the SnF and GO fillers with hydrophobic alkyl groups assisted the dispersion and liquid mixing of the fillers with the SBR. The TGA weight loss curves of the composite samples are shown in Figure 5. The plateau values at a temperature higher than 500 °C indicate the filler content. According to the TGA, the GO and functionalized GO content is approximately 3.5 wt.% and the overall SnF and GO–DA content is approximately 19.1 wt.%. Both plateau values are in good agreement with the filler loadings, which were 4 wt.% for the GO fillers ad 15 wt.% for the silica nanofibers. The TGA thermograms at temperatures lower than 250 °C clearly show that all samples are thermally stable. Thermal degradation is not expected during the mechanical mixing of the composites at 110 °C.

**Figure 6:**
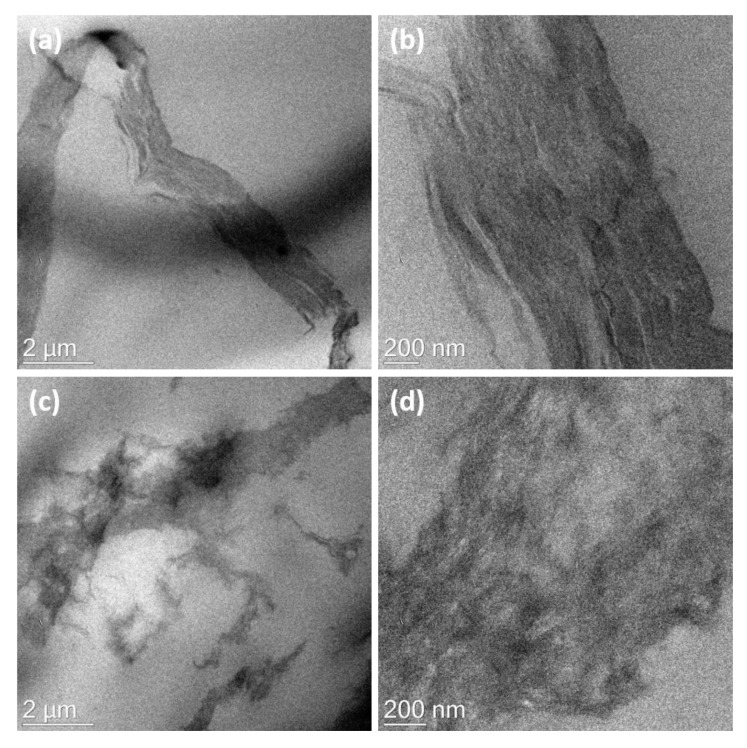
TEM images of the (**a**,**b**) SBR/GO and (**c**,**d**) SBR/rGO–SH.

**Figure 7:**
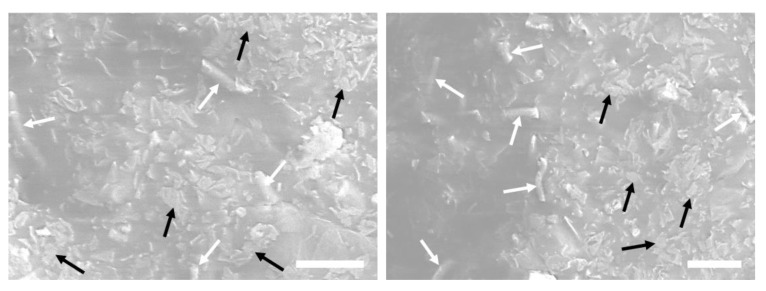
SEM images of the SBR/silica nanofibers (SnFs)/GO–DA composite cross-section. The white arrows indicate the SnFs, whereas the black arrows indicate the GO nanoplatelets. The scale bars are 2 micrometers.

Representative TEM and SEM images of the composites are shown in Figure 6 and Figure 7, respectively. The mechanical mixing was not adequate to exfoliate the GO and rGO–SH platelets in the SBR matrix. Aggregates of the assembled GO and GO–SH layers are shown in Figure 6. Similar results were obtained from the SEM images of the SBR/GO cross-section (not shown here) that indicated large GO aggregates. TGA showed a consistent weight percent content for all composites that can be attributed to an even filler distribution through the entire bulk phase of the elastomer. However, a uniform dispersion of the fillers in the nanoscale is imperative to achieve robust polymer–filler interfaces and improve the such as mechanical, electrical, and thermodynamic properties [19,47,48,49,50]. Liquid mixing has shown significant advantages in the exfoliation of the GO layers [51,52]. The SEM images of the SBR/SnF/GO–DA cross-section in Figure 7 show a good dispersion for both fillers. This hybrid filler configuration combines the synergistic improvements of fillers with different geometries and complementary properties. The GO fillers have good interfacial adhesion with the styrene elastomer and can provide mechanical reinforcement as well as increase the thermal conductivity of the composite. However, the high surface area of the GO and the strong interaction between the GO layers limit the amount of filler material that can be exfoliated and effectively dispersed into the elastomer without forming large aggregates. In Figure 7, it is clearly shown that the GO nanoplatelets are uniformly dispersed through the entire cross-section even though the GO content is only 4 wt%. The addition of SnFs (15 wt.%) allowed the further reinforcement of the composite while maintaining a good dispersion for both filler materials.

**Figure 8:**
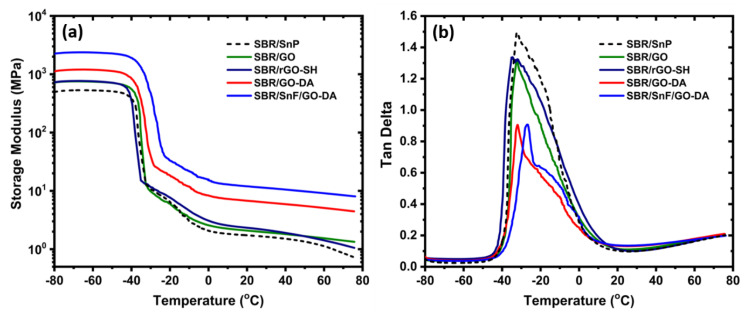
Dynamic mechanical analysis (DMA) plots obtained at 1 Hz for the SBR composites at a broad temperature range (**a**) storage modulus and (**b**) tan*δ* values.

The storage modulus values in the temperature range of −80–80 °C according to the DMA measurements are shown in Figure 8a. The heating rate was 5 °C/min. For comparison, the values of the SBR/SnP composite are also included. The mechanically mixed SBR/GO and SBR/rGO–SH composites show very similar modulus values over the entire temperature range (Figure 8a). At temperatures lower than −40 °C, their storage modulus values increased by approximately 44% compared to that of the SBR/SnP composite. Despite the significantly lower GO content (4 wt.%) compared to the silica nanoparticle content (9 wt.%), the better reinforcement of the GO fillers can be attributed to their higher surface area and their better interfacial adhesion with the SBR. The SBR/GO–DA which was prepared using a liquid mixing technique showed significant improvements compared to the mechanically mixed composites. The storage modulus improved by approximately 60% for temperatures lower than −40 °C. The high temperature plateau values (higher than 20 °C) were also increased by nearly 200%.

The hybrid composite SBR/SnF/GO–DA showed the best reinforcement results. The synergistic improvements between the two different fillers GO–DA and SnF resulted in an approximately 97 and 80% additional increase in the storage modulus for temperatures lower than −40 °C and higher than 20 °C, respectively, compared to the SBR/GO–DA composite. Such high storage modulus values are comparable to those of crosslinked SBR–silica composites (not presented herein). The chemical crosslinking of the composites and the optimization of the crosslinking density requires further investigation and was not studied in this work.

Similar dependencies are also shown in the tan*δ* values of the composites in Figure 8b. The position of the peak maximum, tan*δ_max_*, is associated with the glass transition temperature (*T_g_*). Two relaxation mechanisms contribute to the tan*δ* of the composites. The main peak which is around −32 °C for all composites, except for the hybrid composite where the tan*δ_max_* is shifted to approximately −27 °C, is associated with the *T_g_* of the bulk SBR phase. The second contribution appears as a shoulder at higher temperatures. It can be attributed to a slower relaxation mechanism that is associated with the glass transition of an interfacial SBR layer that is attached to the surface of the fillers and therefore is characterized by slower dynamics [53,54]. This interfacial relaxation process is typical for composite polymeric systems and can be a measure of the polymer–filler interfacial strength. A strong polymer–filler interaction will result in a polymer interfacial layer that is adsorbed (less mobile) on the filler surface and therefore to a robust polymer–filler interface. The composite with the highest interfacial *T_g_* is the SBR/SnF/GO–DA followed by the SBR/GO–DA and SBR/rGO–SH. This interfacial relaxation is also evident in the storage modulus as the slope change in the modulus values in the temperature range from −30 to 0 °C, that is following the abrupt decrease in the modulus values due to the glass transition mechanism (in the temperature range from −40 to −30 °C). The shift of the *T_g_* towards higher temperatures indicate an improved interfacial adhesion between the SBR and the surface of the fillers due to their better dispersion. This is also shown in the intensity of the tan*δ* peak. The tan*δ_max_* values are lower for the SBR/SnF/GO–DA and SBR/GO–DA composites due to the less mobile SBR layer that is adsorbed on the surface of the fillers [55]. The maximum value as well as the integrated area of the tan*δ* peak are associated with the volume fraction of the adsorbed SBR [54]. For elastomers that are designed for tire manufacturing, the tan*δ* value at 60 °C is typically used as an indicator for the rolling resistance. The tan*δ* values for all composites at 60 °C are low and range between 0.16 and 0.18.

### 3.2. SBS Composites

**Figure 9:**
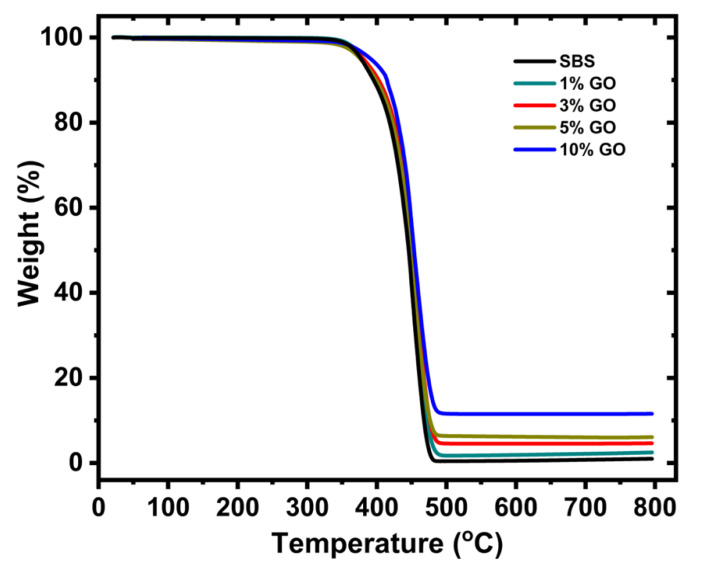
TGA measurements of the styrene–butadiene–styrene (SBS) composites. The GO contents according to the plateau values are in good agreement with the filler loadings which are 1, 3, 5, and 10 wt.%.

**Figure 10:**
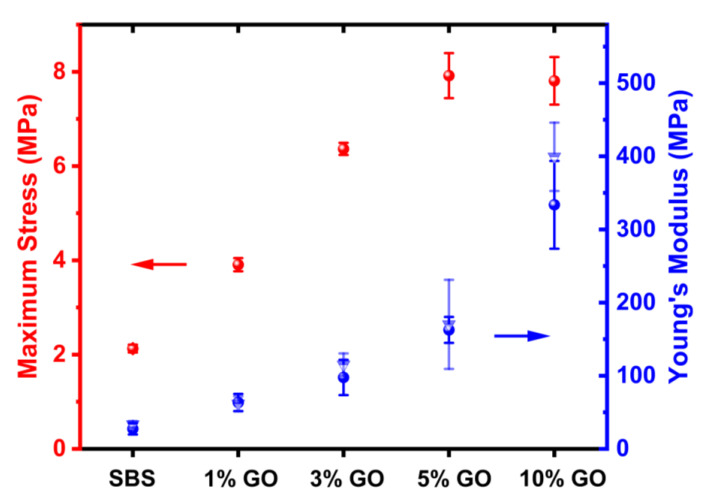
Maximum stress and Young’s modulus values for the SBS composites according to tensile measurements (solid sphere symbols) and nanoindentation measurements (triangle symbols).

SBS composites were prepared at 1, 3, 5, and 10 wt.% GO using a liquid mixing for the GO fillers and the SBS elastomer. The TGA curves are shown in Figure 9. The plateau values are in good agreement with the GO loadings of the composites. The mechanical properties were evaluated using tensile and nanoindentation measurements. The results are summarized in Figure 10. The modulus of the unfilled SBS is approximately 27 MPa which is in good agreement with the results reported in the literature for SBS with similar styrene content [56]. All composites showed notable improvements. A 3.7-fold increase is shown for the maximum stress (before failure) of the 5 wt.% GO composite compared to the unfilled SBS.

Further increase in the GO content did not result in additional improvement and the maximum stress value of the 10 wt.% GO is similar to that of the 5 wt.% GO. This behavior can be associated with the formation of defects due to the GO agglomeration. The 10 wt.% GO composite is characterized by the highest Young’s modulus. A 12-fold increase is shown in the modulus values compared to the unfilled SBS. The tensile modulus values are comparable (within the error bars) with the modulus values that were obtained using nanoindentation measurements. The increase in the modulus values of the composites is almost linear for loadings up to 5 wt.%. An abrupt increase is shown for the 10 wt.% composite which is more than double compared to the modulus value of the 5 wt.% composite. This non-linear behavior can be associated with the increased rigidity (brittleness) of the 10 wt.% composite. The high GO loading increased the amount of the elastomer that is being adsorbed on the filler surface and is thus less mobile. The increased rigidity can improve the abrasion resistance of the elastomer, however the GO loading must be tailored depending on the application in order not to compromise the viscoelastic properties of the composite elastomer.

**Figure 11:**
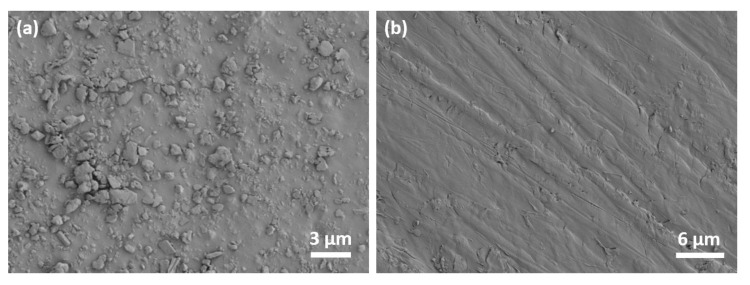
SEM images of the abraded surface of (**a**) the SBS and (**b**) the SBS filled with 10 wt.% GO.

Surface abrasion testing was performed on the unfilled SBS and the 10 wt.% GO composite elastomer using a Taber abrasion tester equipped with two abrading wheels (CS-10) that were rotating on the sample’s surface at 60 rpm. SEM images of the abraded surfaces after 100 abrading cycles are shown in Figure 11. The surface of the unfilled SBS was abraded significantly. Fragments of the elastomer were detached and were agglomerated on the surface. The composite elastomer did not exhibit significant abrasion and only superficial streaks can be observed on the surface. The strong interfacial adhesion between the elastomer and the GO fillers improved the abrasion resistance of the composite elastomer.

### 3.3. Thermal Conductivity

**Figure 12:**
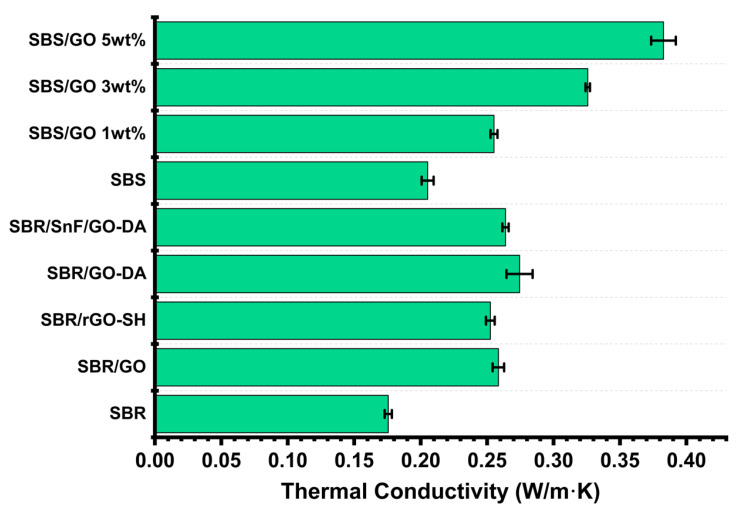
Thermal conductivity values for the SBR and SBS composites.

The thermal conductivity values of the SBR and SBS composites are summarized in Figure 12. The unfilled SBR has the lowest value which is 0.17 W/m·K. Among all SBR composites, the highest increase with respect to the unfilled SBR was for the SBR/GO–DA and was 56%. The hybrid composite showed a slightly lower increase (50%) due to the incorporation of the thermal insulating SnFs. The SBR/GO and SBR/rGO–SH composites that were prepared using mechanical mixing showed a somewhat lower thermal conductivity which were 47 and 44%, respectively. Despite the marked differences in the mechanical properties between the composites prepared by mechanical and liquid mixing (Figure 8), the improvement in the thermal conductivity of the latter is not more than 10%. The filler dispersion and the interfacial adhesion with the elastomer have a significant impact on the reinforcement of the composites. However, the thermal conductivity is more dependent on the formation of percolating paths. The latter can be reached at low weight percent loadings due to the high surface area of the GO fillers even though they are not fully exfoliated. The thermal conductivity of the unfilled SBS is 0.2 W/m·K (18% higher compared to the unfilled SBR). The SBS composites exhibit a similar increase in the thermal conductivity compared to the respective SBR composites. For example, the SBS filled with 3 wt.% GO is characterized by a 59% increase compared to the unfilled SBS, whereas the respective increase for the SBR filled with 4 wt.% GO-DA is 56%. The composite with the highest thermal conductivity is the SBS filled with 5 wt.% GO. Its thermal conductivity value is 0.38 W/m·K and corresponds to a 86% increase compared to the unfilled SBS. The SBS filled with 10 wt.% GO was brittle due to the high GO content and was not studied further.

## 4. Conclusions

Styrene-based elastomer composites with GO and SnF fillers were synthesized. The surface of the GO fillers was modified using two functionalities: cysteamine functionality to introduce sulfur groups and form covalent bonds with the SBR matrix; and dodecylamine functionality to introduce hydrophobic groups and allow the liquid mixing of GO–DA and SBR in toluene. Oxygen reduction took place simultaneously during the cysteamine functionalization. The oxygen content was reduced from approximately 26 at.% in the pristine GO to 16–18 at.% in the cysteamine functionalized GO. The intercalated cysteamine increased the basal plane *d*-spacing of the GO by 4.5 Å. The SBR composites filled with rGO–SH and GO–DA were prepared using mechanical and liquid mixing techniques, respectively. The storage modulus of the SBR/GO–DA composite increased by approximately 60% for the temperatures lower than −40 °C and nearly 200% for the temperatures higher than 20 °C compared to the SBR/rGO–SH. This improvement is associated with the better filler dispersion and therefore the better interfacial adhesion between the GO and the SBR. The latter is also evident by the shift towards higher temperatures of the relaxation mechanism that is associated with the glass transition of the interfacial SBR layer. Synergistic improvements due to the complementary properties of the GO–DA and SnF fillers were achieved in the hybrid SBR/SnF/GO–DA composite. The storage modulus further increased by approximately 97% and 80% for the temperatures lower than −40 °C and higher than 20 °C, respectively, compared to the SBR/GO–DA composite. SBS composites were prepared at 1, 3, 5, and 10 wt.% GO. The maximum stress and modulus values increased almost linearly for the weight loadings up to 5 wt.%. The SBS 5 wt.% GO showed a 3.7-fold increase in the maximum stress and a 6-fold increase in the modulus compared to the unfilled SBS. Further increase in the GO content to 10 wt.% resulted in the increased rigidity of the composite. The increase in the thermal conductivity of the SBR and SBS composites with comparable GO weight percent was similar. The thermal conductivities of the composites were significantly higher than the thermal conductivities of the respective unfilled elastomers. The highest value was 0.38 W/m·K for the SBS filled with 5 wt% GO and corresponds to an 86% increase with respect to the unfilled SBS.

## Figures and Tables

**Table 1:** Surface composition of the GO, rGO–SH-1 (SH-1), and rGO–SH-2 (SH-2) samples according to the core level spectra analysis.

Name	GO at.%	SH-1 at.%	SH-2 at.%
C (sp^2^)	7.9	43.3	39.6
C (sp^3^)	34.7	4.5	8.7
C-O/C-S/C-N	24.2	18.0	19.0
O=C-OH	6.0	1.3	1.8
C=O	0.0	57.7	6.6
O-C	22.3	8.6	11.4
O=C	3.5	9.5	5.0
S-O	0.2	0.5	0.5
H-S-C	0.0	3.4	3.7
N-Aniline	0.0	2.5	2.5
N-Aniline+	0.0	0.7	0.6
Si-O	1.2	2.1	0.8
